# An Enhanced Method for Left Bundle Branch Area Pacing Lead Extraction Using Continuous Femoral Pigtail Countertraction

**DOI:** 10.3390/diagnostics15172198

**Published:** 2025-08-29

**Authors:** Andrei Mihnea Rosu, Theodor Georgian Badea, Florentina Luminita Tomescu, Emanuel Stefan Radu, Maria-Daniela Tanasescu, Eduard George Cismas, Oana Andreea Popa

**Affiliations:** 1 Department of Cardiology, Prof. Dr. Agripa Ionescu Emergency Hospital, 077015 Balotesti, Ilfov, Romania; andrei-mihnea.rosu@drd.umfcd.ro (A.M.R.); radu.emanuel@dcti.ro (E.S.R.); popa.oana@dcti.ro (O.A.P.); 2 Department of Radiology, Prof. Dr. Agripa Ionescu Emergency Hospital, 077015 Balotesti, Ilfov, Romania; theodor.badea@umfcd.ro; 3 Department of Radiology, Carol Davila University of Medicine and Pharmacy, 022328 Bucharest, Romania; luminita.tomescu@umfcd.ro; 4 Department of Semiology, Emergency University Hospital, Carol Davila University of Medicine and Pharmacy, 022328 Bucharest, Romania; 5 Department of Cardiology, Prof. Dr. Theodor Burghele Clinical Hospital, 061344 Bucharest, Romania

**Keywords:** left bundle branch area pacing, lead extraction, cardiac implantable electronic devices infection, continuous lead countertraction, new technique in lead extraction, pigtail tool for lead extraction

## Abstract

**Background:** Left bundle branch area pacing (LBBAP) has emerged as a physiological alternative to conventional pacing, offering improved ventricular synchrony and clinical outcomes. However, extraction of deeply implanted LBBAP leads remains challenging, particularly in the context of device-related infections. **Case Summary**: We report two cases of successful extraction of chronically implanted LBBAP leads using a novel technique based on femoral countertraction with pigtail catheters. In the first case, a deep septal implanted 3830 lead was extracted in a patient with persistent bacteremia and suspected device-related endocarditis. Continuous traction was applied to the mid-portion of the lead using a pigtail catheter introduced via femoral access, facilitating safe removal without the use of powered sheaths proximal to the distal tip of the lead. In the second case, three leads (RA, RV, LBBAP) from a cardiac resynchronization therapy with deffibrilation support (CRT-D) system were completely removed in a patient with device extrusion and pocket erosion, using a dual pigtail approach anchored to the atrial and septal leads. **Results:** In both cases, the technique enabled successful extraction without complications. Procedural times were approximately 70 and 65 min, respectively. In vitro testing suggested that the pigtail catheter applied a sustained moderate traction force (~0.06 kgf), translating to an estimated pressure of 0.85–1.91 kgf/cm^2^ at the septal lead interface. **Conclusions:** This case series demonstrates that LBBAP lead extraction is feasible using a novel femoral countertraction technique with pigtail catheters. Steady, moderate traction over time may provide a safer alternative to forceful subclavicular extraction, especially in chronically implanted deep septal leads. Further studies are warranted to evaluate the reproducibility, safety, and clinical applicability of this approach.

## 1. Introduction

Cardiac pacemakers are implantable electronic devices designed to deliver electrical impulses to the heart when the intrinsic conduction system fails to maintain an adequate heart rate or rhythm [1]. They are commonly indicated in conditions such as symptomatic bradycardia, high-grade atrioventricular block, or certain forms of heart failure requiring cardiac resynchronization [2,3]. Traditional pacing techniques include right atrial pacing, right ventricular apical or septal pacing, and biventricular pacing (used in cardiac resynchronization therapy, CRT) [4,5].

In recent years, physiologic pacing techniques such as His bundle pacing (HBP) [6] and left bundle branch area pacing (LBBAP) have gained attention for their ability to activate the heart’s conduction system more naturally [7].

These newer strategies aim to avoid the dyssynchrony often caused by right ventricular apical pacing and provide more uniform activation of the ventricles [8]. Several observational and randomized studies have reported improvements in ventricular synchrony, left ventricular ejection fraction, and clinical outcomes with LBBAP, particularly in patients with heart failure and left bundle branch block [9].

Despite these advantages, LBBAP implantation is technically more complex and requires specific tools and expertise. Moreover, the long-term management of these leads, especially in the context of device-related infections, is an evolving challenge. The 3830 leads, typically used for LBBAP, are implanted deep in the interventricular septum to engage the conduction system [10]. Their intraseptal course and fibrotic encapsulation over time pose significant obstacles to extraction [11,12]. Lead extraction in the setting of infection is not optional—it is a Class I indication according to major guidelines. However, for LBBAP leads, the experience is limited, and no standardized approach currently exists. Few case reports have described successful retrieval of deeply implanted 3830 leads using powered or mechanical sheaths. These tools, however, carry risks of vascular or myocardial injury, especially when excessive traction is applied. [13].

In this context, we present two cases of LBBAP lead extraction using a novel technique based on continuous femoral countertraction via pigtail catheters. This approach aims to apply steady traction to the lead body, thereby minimizing septal trauma and reducing the need for aggressive proximal dissection.

## 2. Case Presentations

The first case involved a male patient in his 70s with a history of symptomatic atrial fibrillation and prior left bundle branch area pacing (LBBAP) implantation using a SelectSecure 3830 lead [14] at a local hospital approximately 7 years earlier. The patient was admitted to our department with persistent fever and fatigue lasting over 10 days. Medical history included diabetes mellitus and hypertension as well as a prior diagnosis of heart failure with an ejection fraction (EF) of 50%.

**Figure 1:**
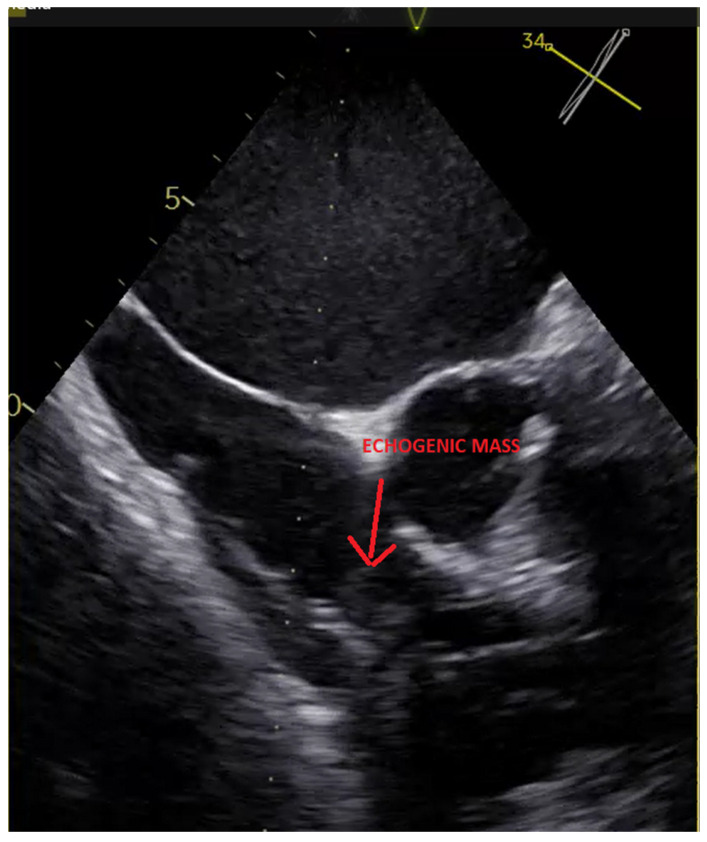
TEE image showing a mobile echogenic mass attached to the pacing lead, consistent with lead-associated vegetation.

The hospitalization was preceded by a recent surgical intervention, complicated by a postoperative infection. Blood cultures grew multidrug-sensitive *Klebsiella* spp., for which the patient received intravenous gentamicin and meropenem for 14 days. Transthoracic and transesophageal echocardiography revealed vegetations as seen in the figure (Figure 1). However, persistent bacteremia raised the suspicion of device-related endocarditis. Given the systemic signs of infection and risk of further complications, complete system extraction, including the deeply implanted LBBAP lead, was indicated.

### 2.1. Extraction Technique

The extraction was performed in an electrophysiology lab under local anesthesia, with cardiovascular surgical backup. The LBBAP lead (Medtronic 3830) [14] had been implanted approximately 7 years earlier. The procedure was initiated with bilateral femoral venous access, which served two purposes: facilitating temporary transvenous pacing during the lead extraction attempts, and providing sheath access for potential vascular complications, such as superior vena cava (SVC) rupture [10]. As one of the 6-F sheaths was not used for pacing or monitoring, we introduced a pigtail catheter through it to engage the mid-portion of the left bundle branch area pacing (LBBAP) lead. This allowed us to maintain continuous countertraction during the procedure. The first step of the intervention included extracting the device from the subcutaneous pocket, followed by careful mechanical dissection of fibrous tissue surrounding the proximal segment of the LBBAP lead.

**Figure 2:**
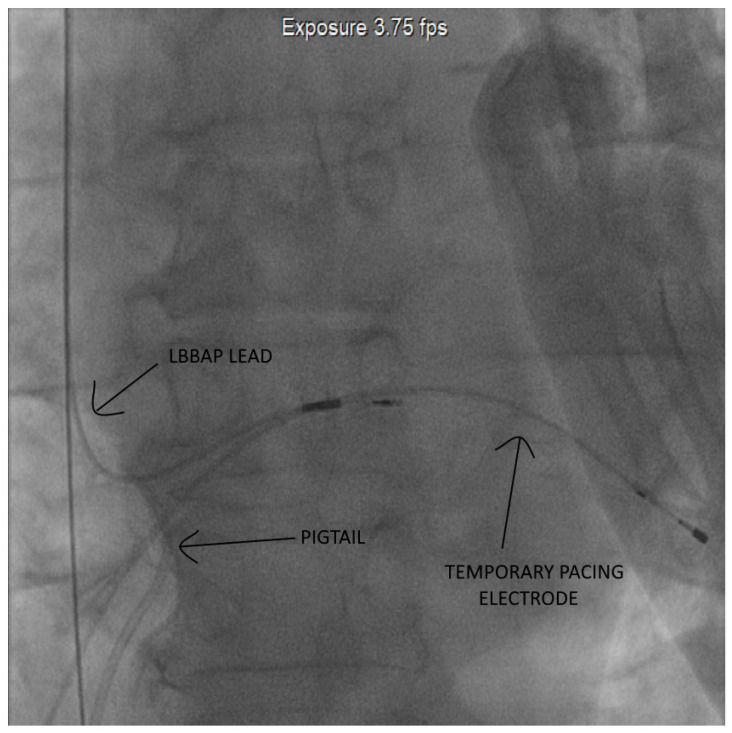
Pigtail is seen anchored to the pacing lead at the level of the left bundle branch area.

**Figure 3:**
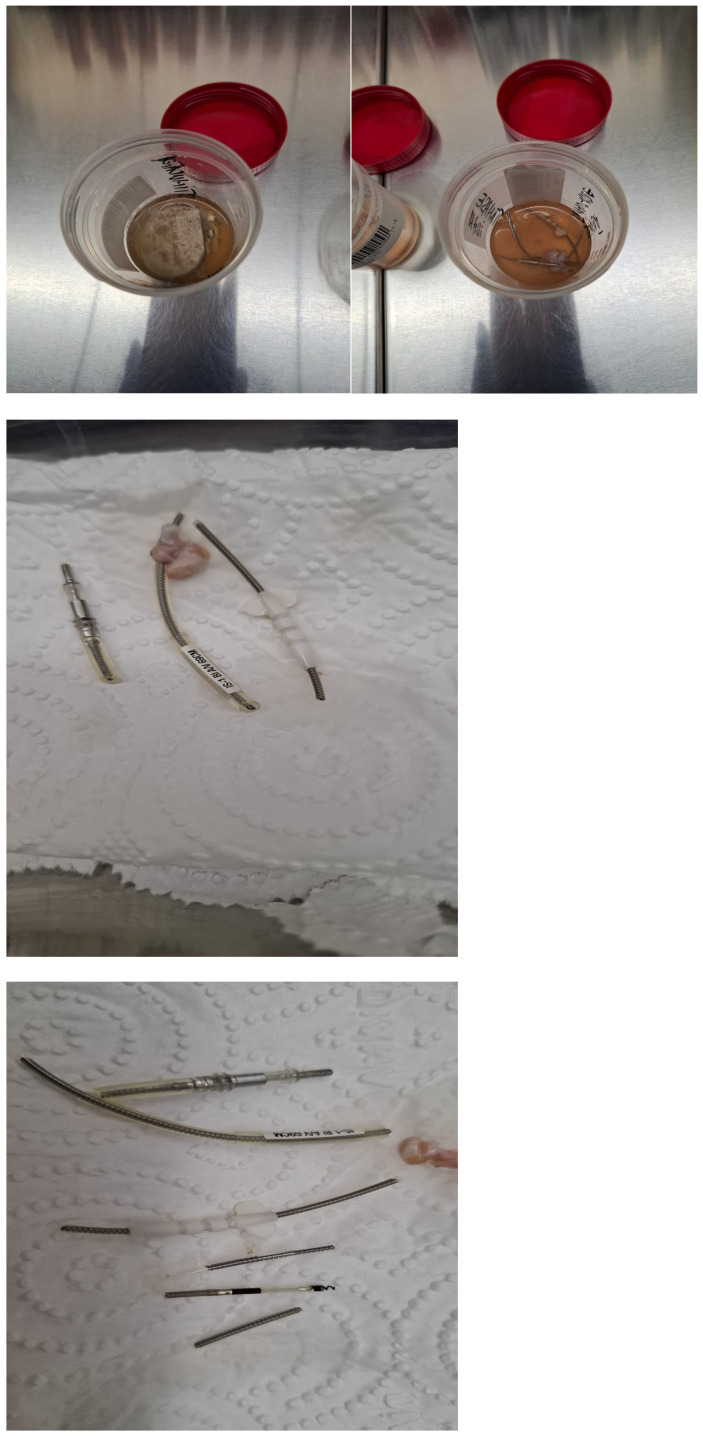
Extracted lead fragments prepared for microbiological analysis, including cultures and further pathogen identification.

As illustrated in the fluoroscopic image (Figure 2), the pigtail catheter was kept under tension at the sheath exit using forceps to ensure stable countertraction. The dissection phase lasted approximately 70 min, after which gentle manual traction was sufficient to achieve complete mobilization and successful extraction of the lead without complications. The entire system was sent for microbiological evaluation which found multidrug-sensitive *Klebsiella* spp. (Figure 3).

The second case involved a 66-year-old patient who presented with a localized generator pocket infection, further complicated by cutaneous erosion and partial extrusion of the device.

The patient had undergone CRT-D implantation five years ago, which included three intracardiac leads: one in the right atrium (RA), one in the right ventricular (RV) apex, and one at the interventricular septum for conduction system pacing (LBBAP with a normal stylet-driven Medtronic lead [14]).

**Figure 4:**
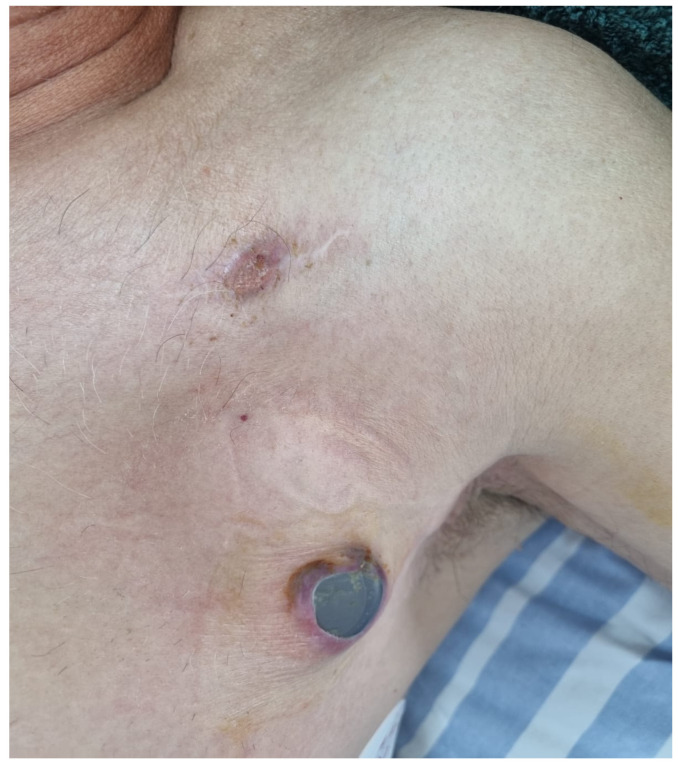
Clinical image showing device extrusion through the skin, with visible displacement of the CRT-D generator and signs of inflammation, including erythema and tissue breakdown at the pocket site.

**Figure 5:**
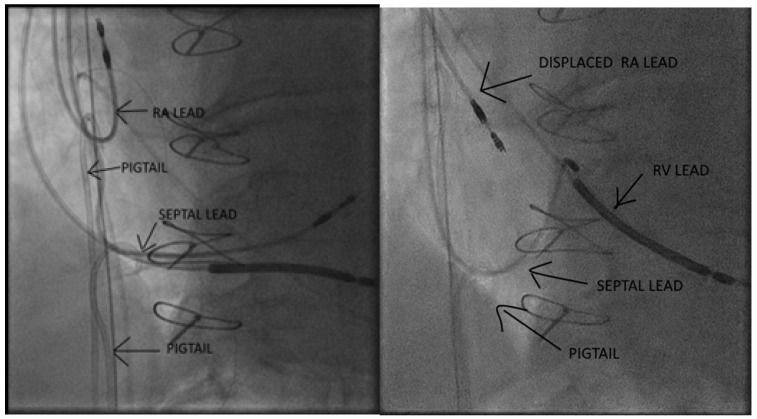
Angiographic images showing pigtail catheter traction applied to the pacing leads followed by progressive displacement of their original implantation sites.

Given the extent of pocket erosion and the elevated risk of systemic infection (Figure 4), complete removal of the device hardware was warranted. In contrast to the first case, the presence of three intracardiac leads necessitated a modified femoral countertraction strategy. Two pigtail catheters were introduced via separate femoral sheaths and sequentially anchored—one to the right atrial (RA) lead and the other to the left bundle branch area pacing (LBBAP) septal lead. Following partial mobilization, one of the catheters was repositioned to engage the right ventricular (RV) lead. This configuration provided stable, distributed countertraction, enabling rapid en bloc extraction of all three leads. The total duration from initial femoral engagement with the pigtail catheters to complete lead removal was approximately 65 min, as estimated from angiographic recordings. No intra- or post-procedural complications were observed. Fluoroscopic images confirmed optimal pigtail positioning and real-time countertraction during extraction (Figure 5).

### 2.2. Postprocedural Course

The patient was monitored in the cardiac intensive care unit, with a temporary lead placement in the right ventricle. No immediate complications, such as pericardial effusion or tamponade, were observed. Blood cultures became sterile after 14 days of targeted intravenous antibiotic therapy. After two weeks in the critical cardiologic care unit, the patient underwent reimplantation of a pacing system on the contralateral side, avoiding deep septal placement.

The 14-day follow-up visit revealed no signs of local or systemic complications, no evidence of inflammatory syndrome, and pacing parameters were within normal functional limits.

Following the procedure, the second case patient was monitored in the cardiac intensive care unit for 48 h. No immediate complications, such as pericardial effusion, tamponade, vascular injury, or arrhythmic events, were observed. Hemodynamic stability was maintained throughout, and no signs of systemic inflammatory response or local pocket complications were noted.

Given the patient’s stable condition, he was transferred back to the referring tertiary center where the initial CRT-D implantation had been performed and a temporary pacing system that had been previously established with an active-fixation lead positioned in the right ventricle and connected to an external VVI pacemaker. This configuration was maintained until reimplantation of a definitive cardiac resynchronization therapy defibrillator (CRT-D) system could be performed under optimized clinical and infectious conditions.

## 3. Discussion

Left bundle branch area pacing (LBBAP) has gained widespread adoption for its physiologic benefits and favorable pacing parameters, including low thresholds, narrow QRS duration, and more synchronized ventricular activation compared to traditional right ventricular pacing. In patients requiring cardiac resynchronization therapy (CRT), LBBAP has emerged as a viable alternative to biventricular pacing, particularly in cases where coronary sinus lead placement is technically difficult or fails to achieve optimal results [15]. However, the increasing uptake of LBBAP has introduced a new challenge: the safe and effective extraction of deeply embedded septal leads. Unlike traditional RV leads that typically follow a relatively straight path and are anchored within the trabecular myocardium, LBBAP leads—especially Medtronic 3830 models—are screwed deep into the interventricular septum, often close to or within the subendocardial conduction tissue. Over time, fibrous encapsulation, calcification, and endothelialization around the distal portion of the lead can significantly increase extraction difficulty [7].

Previous reports have documented extraction of LBBAP leads using powered or mechanical sheaths, such as rotational cutting sheaths or telescoping systems. While effective, these approaches carry non-negligible risks of myocardial perforation, septal damage, and even fatal complications such as tamponade [16,17]. Furthermore, aggressive proximal traction can result in lead breakage, incomplete extraction, or tissue avulsion, especially in chronically implanted leads [18,19]. The technique described in our report represents a conservative, alternative strategy based on biomechanical principles. By applying continuous, low-force countertraction to the mid-portion of the lead via a femoral pigtail catheter, we hypothesize that the cumulative force exerted over time facilitates gradual detachment of the fibrotic attachments. The estimated traction force of 0.06 kgf, translating to a septal interface pressure of ~0.85 kgf/cm^2^, appears sufficient to promote safe extraction without generating shear stress or abrupt displacement. This “slow tension” concept mirrors the principles used in orthopedic traction or tissue expansion, where biological structures are gently mobilized over extended periods.

Another advantage of this approach is its versatility. In the second case presented, we successfully adapted the method to extract multiple leads (RA, RV, and LBBAP) by distributing tension across multiple pigtail loops. This configuration provided balanced traction without the need for powered tools or aggressive dissection from the subclavicular approach. Notably, no complications such as pericardial effusion, vascular injury, or lead fracture occurred in either case.

To our knowledge, this is the first published description of a pigtail-based continuous countertraction technique specifically applied to LBBAP lead extraction. While our experience is limited to two cases, the results suggest that this method may be reproducible and potentially safer in selected patients, especially those with infected systems where lead preservation is not an option. It may also be applicable in situations where specialized extraction tools are unavailable or contraindicated due to comorbidities. Therefore, we conducted preliminary in vitro tests to estimate the force and corresponding pressure exerted by a pigtail catheter loop positioned around the lead’s mid-portion. These tests considered a simplified mechanical model in which one end of the lead was fixed at the subclavicular insertion point (simulating fibrous anchoring), while the other was embedded within septal tissue, the key area of interest.

**Figure 6:**
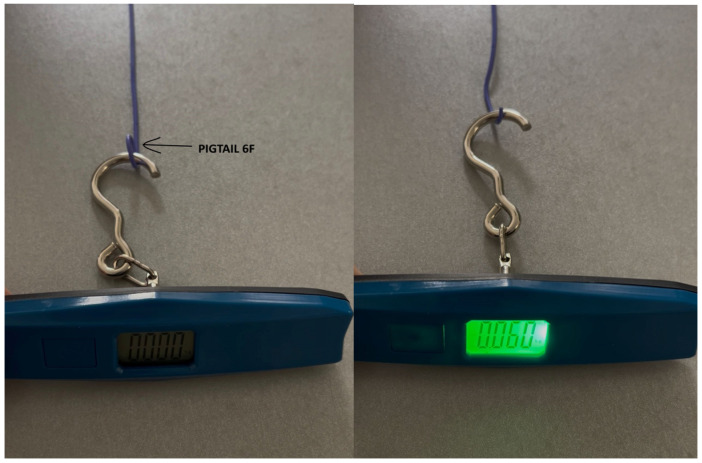
The figures show the twisted tip of the pigtail catheter suspended from a scale, illustrating the force applied at a single contact point. Figure left—without traction; figure right—with traction.

As demonstrated in Figure 6, the pigtail catheter was able to maintain a traction force of approximately 0.06 kgf. When translated to the cross-sectional area of a 5F lead (approximately 0.0314 cm^2^), this corresponds to a pressure of 1.91 kgf/cm^2^ applied to the body of the pacing lead. Taking into account dissipation of force at both anchoring points, we estimate a local pressure of approximately 0.85 kgf/cm^2^ at the septal contact site. Frictional forces along the lead path were not included in the calculation, as the intraseptal trajectory is typically linear and lacks significant curves that would introduce major frictional resistance.

Considering the total procedural time of 70 min, we propose that prolonged application of moderate traction via a pigtail loop may represent a viable and safer alternative to short-duration, high-force extraction techniques. This method may be particularly useful in future cases where deep septal lead anchoring is encountered.

Moving forward, larger prospective studies or registry data will be essential to validate this technique. Standardized measurement of traction force, procedural times, complication rates, and long-term outcomes should be included in future research. Furthermore, comparative studies between traditional extraction methods and the pigtail technique may clarify its relative advantages or limitations.

Infections involving cardiac implantable electronic devices (CIEDs) continue to pose significant clinical challenges, and prompt complete hardware removal remains the cornerstone of effective management [20]. Delays in extraction are associated with increased morbidity and mortality [21]. Therefore, any technique that facilitates safer and faster removal—particularly of unconventional leads such as those used in LBBAP—represents an important step forward in clinical electrophysiology.

## 4. Conclusions

Extraction of deeply implanted LBBAP leads is technically feasible and should be pursued without delay in patients with device-related infections. Careful planning, appropriate tools, and experienced personnel are essential to ensure procedural success and patient safety.

This case highlights both the clinical urgency and the technical feasibility of extracting a left bundle branch area pacing (LBBAP) lead in a patient presenting with persistent bacteremia. In the context of sepsis, especially with positive blood cultures and no other clear source of infection, timely removal of all potentially infected hardware—including pacing leads—is essential to prevent serious complications such as endocarditis, septic emboli, or systemic dissemination. Delayed extraction in these scenarios is associated with increased morbidity and mortality.

From a technical standpoint, our approach involved the application of steady and controlled traction to the mid-portion of the lead using a pigtail catheter loop introduced via femoral access. This strategy allowed us to minimize the need for excessive force at the subclavicular entry site, which can be associated with tissue trauma and complications such as myocardial avulsion or vascular injury.

Preliminary in vitro testing showed that the pigtail catheter exerted a traction force of approximately 0.06 kgf, corresponding to a pressure of 1.91 kgf/cm^2^ on the lead surface, and approximately 0.85 kgf/cm^2^ at the level of the septal fixation zone. These values suggest that prolonged moderate traction may be sufficient to disengage the lead from the myocardium without the need for high-force maneuvers, especially when used over an extended operative time, such as the 70 min required in our case.

While limited to a single experience, this technique may offer a safer alternative in similar cases, particularly when conventional traction methods are deemed risky. Further investigation is required to validate this method and to determine whether it can be adopted as a standardized approach for LBBAP lead extraction in infected patients.

Our findings are consistent with recent multicenter and single-center studies evaluating conduction system lead extraction (Table 1). Vijayaraman et al. [22] reported high procedural (99%) and clinical (100%) success rates in 341 patients, with a low need for advanced extraction tools, particularly for leads with a dwell time under 3 years. Migliore et al. [23] confirmed the feasibility and safety of extraction even for leads implanted for more than a decade, with a 94% complete extraction rate and no major complications. Data from Wagner et al. [24] are still limited in abstract form but support the overall notion of high success and low complication rates.

These results suggest that left bundle branch area pacing (LBBAP) lead extraction is technically feasible and safe, with procedural characteristics largely dependent on lead dwell time rather than lead location. Manual traction appears sufficient for leads implanted for less than a year, whereas mechanical sheaths are more often required for older implants. Major complications remain rare, and re-implantation is feasible in most cases.

## Figures and Tables

**Table 1:** Detailed study summary.

Study (Citation)	Design and Population	Lead Type and Dwell Time	Extraction Tools Used	Success and Outcome	Complications and Notes
Vijayaraman et al. [22]	Retrospective, international multicenter (10 centers), with 341 patients.	224 His-bundle, 117 LBBAP; mean dwell 22 ± 26 mo.	Manual (most); mechanical in 10% (>6 mo), laser in 3%, femoral tools in 3 cases.	Complete procedural success 99%; clinical 100% (3 patients retained fragments); re-implantation success 95%.	Minor complications in 2.1%; low need for advanced tools; dwell mostly <3 yrs.
Migliore et al. [23]	Single-center, 6 patients, 17 leads (Italy); mixed His/LBBP.	3830 lumenless leads; mean dwell 97 ± 90 mo (8–193 mo).	Manual successful in 2; evolution RL mechanical sheath in others.	94% complete extraction; 1 minor remnant; no major complications; median follow-up 15 ± 13 mo.	One femoral AV fistula; no conduction damage or septal defect.
Wagner et al. [24]	Multicenter observational (cited in *Heart Rhythm* abstract).	Likely His + LBB leads; details not in abstract.	Not specified in summary.	Not specified—full text needed.	Not tracked in abstract; presumably similar outcomes.

## Data Availability

Data are contained within the article.

## Abbreviations

The following abbreviations are used in this manuscript:

CIEDs	Cardiac implantable electronic devices
CRT-D	Cardiac resynchronization therapy with deffibrilation therapy
EF	Ejection fraction
LBBAP	Left bundle branch area pacing
HPB	His bundle pacing
TEE	Trans-esophageal echo-cardiograph
RA	Right atrium
RV	Right ventricle
SVC	Superior vena cava
VVI	Unicameral pacemaker
